# ECC at LLETZ—An Exploratory Retrospective Cohort Study

**DOI:** 10.3390/jcm13226671

**Published:** 2024-11-06

**Authors:** Chiara Paternostro, Elmar A. Joura, Johannes Ott, Stefan Ghobrial, Eva M. Langthaler, Sophie Pils

**Affiliations:** 1 Department of Obstetrics and Gynecology, Division of General Gynecology and Gynecologic Oncology, Medical University of Vienna, 1090 Vienna, Austria; 2 Department of Obstetrics and Gynecology, Division of Gynecologic Endocrinology and Reproductive Medicine, Medical University of Vienna, 1090 Vienna, Austria; 3 Department of Pathology, Medical University of Vienna, 1090 Vienna, Austria

**Keywords:** ECC, high-risk HPV, LLETZ, adequacy, squamous epithelial cells, transformation zone, HSIL, margin status

## Abstract

**Background:** The aim of this study is to evaluate the distribution of the histological results of the endocervical curettage (ECC) at large loop excision of the transformation zone (LLETZ) as well as the additional diagnostic information and its impact on further clinical treatment recommendations in accordance with national guidelines. The ECC in addition to LLETZ can serve to detect (pre)cancerogenic lesions above the endocervical cone margin, although its predictive value as well as diagnostic accuracy remains vague due to limited studies performed on this topic. **Methods:** In this retrospective cohort study, 1121 patients who underwent LLETZ and concomitant ECC during a twelve-year period (2009–2021) were analyzed. The main outcome parameters were the histological diagnosis and incidence of adequate or inadequate ECC specimens. **Results:** In 1.7% of the specimens, ECC performed at the completion of LLETZ yielded additional diagnostic information. The histological result of the ECC had an impact on subsequent therapeutic approach in 2 of the 1121 patients (0.2%). Furthermore, a negative ECC yielded a low negative predictive value (NPV) of 43.8% for the detection of residual disease. **Conclusions:** As current guidelines increasingly support the performance of an HPV test six months after LLETZ as an indicator of treatment success irrespective of the margin status, the routine performance of an ECC at LLETZ remains questionable.

## 1. Introduction

Excisional biopsy, especially the large loop excision of the transformation zone (LLETZ), is an established therapy to remove cervical intraepithelial neoplasia (CIN) and thus reduce the risk of cervical cancer [1,2,3,4,5,6].

Predictive parameters for treatment failure (i.e., residual disease) such as positive endocervical cone margins or persistent high-risk human papillomavirus (hr-HPV) infection have been identified [7,8,9,10,11,12]. Immediate surgical treatment as a consequence of an incomplete margin resection is recommended in cases with suspected residual glandular (i.e., adenocarcinoma in situ, AIS) or invasive disease, whereas conservative follow-up with obligatory hr-HPV testing and cytology after six months is recommended in patients with high-grade squamous intraepithelial lesion (HSIL) [13,14,15,16]. However, if negative margins have been achieved without evidence of an invasive disease, postoperative follow-up including an HPV test and cytology is also recommended after 6 months [17,18,19,20]. 

An endocervical curettage (ECC) in addition to LLETZ can serve to diagnose (pre)cancerogenic lesions above the endocervical cone margin, although its predictive value as well as diagnostic accuracy remains vague on account of limited studies performed on this topic [21,22,23,24]. In accordance with the physiological anatomy of the cervix, ECC specimens should contain glandular epithelium of the endocervix and may, in addition, contain squamous epithelium of the ectocervix. Hence, in the case of the absence of epithelial cells of the transformation zone, the technical performance of ECC may have been incorrect and therefore the specimens are considered as inadequate [25,26].

Therefore, the clinical utility of an ECC at the time of LLETZ is controversial, not only because there are no standardized reporting models for ECC specimens such as the Bethesda System for cervicovaginal cytology, which includes a clear definition of specimen adequacy based on various factors like the presence of the transformation zone (TZ) components or minimum threshold of cells, but also due to the limited number of published studies evaluating the additional diagnostic benefit of ECC, resulting in a lack of uniform guidelines [24,27,28,29].

The aim of this study is to evaluate the additional diagnostic information of the ECC following LLETZ and its impact on further clinical treatment recommendations in accordance with national guidelines.

## 2. Materials and Methods

### 2.1. Patient Population and Study Design

In this retrospective single-center cohort study, 1175 patients who underwent LLETZ and concomitant ECC for cervical dysplasia at the Department of Obstetrics and Gynecology, Medical University of Vienna, during the period of January 2009 to December 2021 were included. Due to the diagnosis of invasive cancer in the LLETZ specimens, 54 patients had to be excluded and therefore a total of 1121 patients were analyzed. 

ECC was performed as part of the routine procedure and directly after removing the cervical cone in our tertiary care center with a Kevorkian curette, which enabled us to perform this retrospective study with a large number of cases. After removing the cervical cone and performing an ECC, a coagulation of the cervical wound bed was routinely undertaken. The histological results of the ECC specimens were divided into the following three main groups: negative ECC (i.e., adequate specimen with epithelial cells of the TZ such as glandular and squamous cells), positive ECC containing neoplastic epithelium cells, and inadequate specimens. The latter were defined either by the presence of only endometrial cells or by unsatisfactory samples without cells for histologic interpretation.

Cone margin status was divided into the following three categories: non-evaluable margin, negative margin, or positive margin [30]. Positive margins were reported as endocervical or ectocervical, and specimens with both positive endo- and ectocervical margins were also assigned to the subgroup of positive endocervical margins. Histological results were divided into low-grade intraepithelial lesion (LSIL), HSIL, and adenocarcinoma in situ (AIS). Within the study period, specimens were examined and graded by eight pathologists specialized in the field of gynecological pathology. 

TZ was reported as type 1 (squamocolumnar junction completely visible), type 2 (squamocolumnar junction partially visible), and type 3 (squamocolumnar junction not visible) [20]. Hr-HPV genotypes were diagnosed using the cobas^®^ HPV test and described individually (HPV 16 and 18) or in a pooled category which was reported as “other hr-HPV” (HPV 31, 33, 35, 39, 45, 51, 52, 56, 58, 59, 66 and 68). Immediate treatment was defined as surgical intervention within 6 months. 

“Additional diagnostic information” refers to either specimens with LSIL/HSIL/AIS in the ECC but negative endocervical cone margins or the detection of invasive disease only in the ECC. “Impact on therapeutic approach” refers to a modification of the subsequent therapeutic approach due to the ECC result according to recent guidelines.

### 2.2. Parameters Analyzed 

Relevant data were extracted retrospectively from the medical records. The main outcome parameters were the histological diagnosis and incidence of adequate or inadequate ECC specimens. In addition, the following clinical characteristics were described: TZ type, hr-HPV status, age of the patient at LLETZ, histological result of the specimen (LLETZ and ECC), incidence of immediate surgical treatment, and histological diagnosis of repeated conization (re-LLETZ) or hysterectomy (HE). 

### 2.3. Statistical Analysis

**Figure 1:**
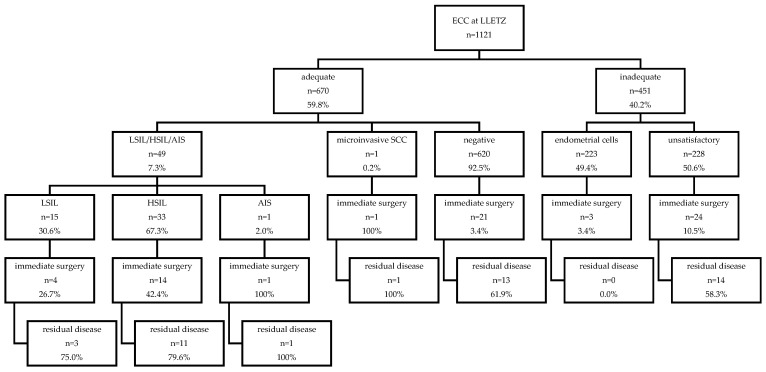
Overview of histological results of ECC at LLETZ and follow-up data of residual disease according to ECC result. Abbreviations used: ECC, endocervical curettage; LLETZ, large loop excision of the transformation zone; LSIL, low-grade squamous lesion; HSIL, high-grade squamous lesion; AIS, adenocarcinoma in situ; SCC, squamous cell carcinoma.

Nominal variables are reported as numbers and frequencies, and continuous variables are reported as medians and interquartile ranges (IQRs). Statistical analyses were performed with SPSS v26 (released 2019, IBM Corp., Armonk, NY, USA) using the Mann–Whitney U test or Pearson’s chi-squared test, where appropriate. A bivariate analysis was performed in order to evaluate the effect of the histological result, margin status, HPV status, and ECC on the occurrence of persistent disease detected by subsequent surgical treatment after LLETZ. A two-sided *p* value < 0.05 was considered significant. Figure 1 was generated using Microsoft Word (version 16.43). 

## 3. Results 

A total of 1121 patients who underwent LLETZ were analyzed. The median age at the time of LLETZ was 36 years (IQR 31–44), and HPV 16 was found most frequently (Table 1). 

A majority of the patients had a TZ 1 (57.9%), and positive margins were observed in 24.4% (n = 276/1121), with immediate surgical treatment performed in 68 patients. 

Up to 50 of the 1121 ECC specimens (0.04%) showed a positive result (15 LSIL, 33 HSIL, 1 AIS, 1 microinvasive carcinoma; Figure 1), whereas 620 were defined as negative. Inadequate ECC specimens were found in 451/1121 cases (40.2%), of which 223 contained only endometrial cells and 228 were unsuitable for analysis as they did not contain epithelial cells. 

Negative cone margins were found in 74.5% of the LLETZ specimens (n = 835/1121), of which 1.3% (n = 11/835) had a positive ECC. In 168 cases, the endocervical margin was found to be positive with a positive ECC in 32 cases (19.0%), negative in 79 cases (47.0%), and inadequate in 57 cases (33.9%). Ectocervical margins were involved in 108 LLETZ specimens, with a positive ECC in 6 (5.5%), negative in 60 (55.6%), and inadequate in 42 cases (38.9%). Non-evaluable margins were described in ten LLETZ specimens, of which five ECC samples were detected to be negative, four inadequate, and one with evidence of HSIL. 

Overall, positive ECC samples were significantly more likely to be detected in LLETZ with involved or non-evaluable margins compared to clear margins (n = 39/286 vs. n = 11/835; *p* < 0.001).

In addition, the histological result of the ECC provided additional diagnostic information of the margin status in 19 cases (1.7%), of which two patients would have been counseled with a different therapeutic approach according to the current national guidelines due to the ECC (Table 2). It is noteworthy that one of these patients had no evidence of neoplastic cells in the LLETZ specimen but exhibited evidence of HSIL in the ECC sample (Table 2). No further surgical treatment was performed as the patient became pregnant shortly after LLETZ, but postpartum follow-up showed no evidence of HSIL as assessed by cytology and a negative HPV test.

In the other case, the ECC detected evidence of microinvasive squamous cell carcinoma (SCC) whereas the LLETZ specimen exclusively identified HSIL. In particular, the patient was considered high risk due to immunosuppression following organ transplantation and concomitant HPV 18 infection. The final histological result through hysterectomy showed an adenosquamous carcinoma with the origin of the endocervix.

Of the 1121 study subjects, 68 patients received immediate surgical treatment due to involved margins with the confirmation of residual disease in 61.8% (n = 42/68). The sensitivities and specificities of prognostic values for the detection of persistent cervical disease are summarized in Table 3.

The highest sensitivity to predict residual disease was observed for margin positivity (90.5%), whereas the highest specificity was found in cases with a negative ECC specimen (80.7%; Table 3). In addition, a bivariate analysis was performed, and it showed a significant association between persistent disease and HPV 16 or 18 infection with an OR of 4.80 (CI 1.11–20.76, *p* = 0.026), whereas the other studied parameters—notably including the performance of an ECC at the completion of LLETZ (OR 2.33 (CI 0.73–7.46); Table 4)—showed no statistically significant result. 

## 4. Discussion

In this single-center retrospective cohort study, the clinical impact of 1121 ECC specimens performed at the completion of LLETZ was assessed, resulting in 1.7% of the ECC samples revealing additional diagnostic information and 0.2% influencing the subsequent therapeutic approach according to the study design. Although there is no international consensus on post-treatment surveillance strategies yet, a vast number of cross-national guidelines support the performance of HPV testing six months post-LLETZ, which is known as the most accurate predictor of treatment outcome, irrespective of the margin status [31,32]. A meta-analysis published by Arbyn M. et al. showed that a positive margin status only detects 56% of persistent/recurrent disease, whereas HPV testing diagnoses 91%, which raises the question of whether it is still clinically necessary to differentiate follow-up strategies according to margin status alone [7,11]. Even though positive margins were found to have a five times higher relative risk of persistent/recurrent HSIL compared to negative margins, short-term follow-up surveillance after LLETZ may still not need to be modified based on resection margins, since a PPV of 95% for the absence of residual disease with the clearance of hr-HPV after LLETZ was observed [3,33]. 

According to the national guidelines, which were applied retrospectively on the studied collective, a follow-up surveillance interval of 6 months is recommended, irrespective of the margin status [11,15]. Therefore, the additional information obtained by the ECC (n = 19) altered further treatment counseling in two women (0.2%) as follows: one case of microinvasive SCC in the ECC but only HSIL in the LLETZ specimen and one HSIL in the ECC but a negative LLETZ specimen (Table 2). Endocervical residual disease was suspected exclusively due to the ECC in 17 women without further clinical impact on therapeutic counseling under the terms of the study design. 

In the analyzed collective, the ECC as a prognostic parameter for persistent disease showed a sensitivity of 35.7% and specificity of 80.7% (Table 3). Previous reports have found a sensitivity rate of an ECC at LLETZ ranging from 38.0% to 77.8% and a specificity rate from 55.6% to 97.5%, although the existing data are limited on this topic [34,35,36]. Consequently, a positive ECC indicates a high probability of persistent disease, with a PPV of 75.0% (Table 3). Conversely, our data suggest that a negative ECC may not be considered a reliable diagnostic parameter due to its relatively low sensitivity and NPV of 43.8%. Furthermore, there was no statistically significant association between the ECC result and persistent disease (OR 2.33 (CI 0.73–7.46); Table 4). On the other hand, performing the ECC during a colposcopy prior to LLETZ enhanced the overall detection rate of high-grade lesions by an increase of 7.6% to 16.7%, as evaluated by van der Marel et al. [37]. This percentage varies within the literature and ranges from 1% to 17% depending on various factors such as age and TZ type [38,39]. Ruan et al. described that the general sensitivity and specificity to detect HSIL by colposcopy is 59.3% and 93.8%, respectively, while specific percentages concerning sensitivity and specificity related solely to an ECC at a colposcopy are lacking [38,40]. In contrast, the diagnostic role of the ECC performed 12 months after LLETZ has been evaluated by Schneider et al., exhibiting a sensitivity of 38% and a specificity of 85% for the ECC specimens [36]. The sensitivity and specificity of post-LLETZ HPV testing predicting residual disease was found to be considerably higher, with 91% and 84%, respectively [32]. 

In 59.8% of the cases studied, the ECC specimens contained components of the TZ and were therefore referred to as adequate. It is notable that the incidence of inadequate ECC samples due to a lack of TZ components varies in the literature. While Suzuki et al. found 24.8% of ECC samples to be inadequate, which is considerably less than in this analysis (40.2%), a similar rate of positive ECC samples (7.9%) was described, which is comparable to our findings (7.3%) [21]. Since there are no standardized guidelines for specimen adequacy of an ECC, the definition used in this analysis was limited to the type of epithelial cells found in the specimen. Beyond that, other quality indicators such as the definition of minimum cellularity in order to detect an HSIL on an ECC are discussed in the literature. A published study of Alqabbani et al. suggested that less than 1000 squamous cells in ECC specimens should be considered as inadequate, whereas a cellularity of more than 10,000 squamous cells is adequate to detect an HSIL [25]. Thus, a specimen consisting of a low cellularity without evidence of neoplastic cells might be described as “inadequate” rather than “negative”, which would lead to a higher number of clinically ineligible samples but could also improve the diagnostic accuracy of the ECC. 

A positive ECC was detected significantly more often in patients with positive than with negative margins (13.6% vs. 1.3%; *p* < 0.001), which is in alignment with the findings of Cuello et al. [41]. 

On the other hand, the presence of suspected AIS demands a different therapeutic approach, with hysterectomy as the preferred therapy, even though fertility sparing management for patients with a wish to conceive is possible but may only be justifiable if negative margins can be achieved [3]. Due to the frequent origin of AIS in the endocervical canal and the higher risk of being multifocal, i.e., skip lesions, an ECC can help to detect occult or remaining disease in addition to LLETZ in cases of suspected glandular disease [42,43]. Nevertheless, clear clinical recommendations on whether to use an ECC at the time of LLETZ in suspected glandular disease are lacking, and the available data are inconclusive. A study published by Kietpeerakool et al. showed that ECC alone was not useful in detecting residual AIS after excisional treatment since approximately 40% of the women who were diagnosed with residual disease on a subsequent surgery had a negative ECC result [30]. This observation is also supported by our data indicating that none of the ECC specimens contained neoplastic cells when residual AIS was diagnosed by subsequent surgery. In contrast to these findings, other reports have described the utility of an ECC in case of AIS, such as the study of Lea et al. which identified a PPV of 100% for an ECC in predicting residual AIS [22].

The recently published retrospective study of Scherer-Quenzer et al. detected a rate of 8.4% of specimens with HSIL at the endocervical margin as well as in the ECC and stated a significantly higher risk for recurrent dysplasia than with HSIL only at the endocervical margin but a negative ECC [44]. These findings differ from our analyzed cohort, in which only the occurrence of HPV 16/18 showed a significant result as a risk factor for persistent disease with an OR of 4.80 (1.11–20.76; Table 4). These divergent findings may be attributed to the different study protocol and follow-up period as well as number of included cases (404 vs. 1121). As the follow-up period in this present study was 6 months, recurrent disease, which may occur 12 months or more after LLETZ, could not be evaluated. Nonetheless, additional clinical studies are necessary to gain a more comprehensive understanding of the clinical impact of ECC during LLETZ.

In addition, the NHS cervical screening program (CSP) specifies a further subgroup that may receive a distinct follow-up counseling according to margin status, namely women ≥50 years of age with a TZ 3. When residual CIN 3 is suspected at the deep lateral and/or endocervical resection margin, the performance of a subsequent surgical intervention is advised [16]. Hence, in this very specific subgroup, performing an ECC at the time of LLETZ can be warranted, as the margin status may actually lead to a direct clinical and surgical consequence. In this analysis, three patients of this specific subgroup with clear margins but a positive ECC were detected. According to the NHS CSP guideline, the ECC result in these three cases would therefore also have had a clinical impact on further decision making. As the management of this subgroup is not consistently mentioned in other cross-national guidelines, we chose to not include this factor in the data analysis. 

Even so, the utility of a routine ECC remains a topic of debate concerning its use during a colposcopy. Current international guidelines are offering varied recommendations, with most of them advising selective rather than comprehensive application of an ECC at a colposcopy. Massad et al. published detailed statements in “Colposcopy Standards: Guidelines for ECC at Colposcopy” such as recommendations to use an ECC in all cases of a TZ 3, a known positive test for HPV 16 or 18, and in patients previously treated for intraepithelial lesions [45]. The published guidelines of the World Health Organization (WHO) on cervical cancer prevention and control indicate the specific circumstances to use ECC such as suspected precancerous lesions hidden in the cervical canal, glandular lesions revealed by a PAP smear or women with a TZ 3 [46]. Another consensus statement published by the “American Society for Colposcopy and Cervical Pathology (ASCCP)” is also rather in favor of a selected use of an ECC at a colposcopy and, furthermore, specifies its clinical utility for short-term follow-up six month after treatment for example if HPV screening is not available [11]. Even though the guidelines mentioned do not explicitly address their recommendations of an ECC at the completion of LLETZ, some points could be taken into account. As shown in Table 4, an HPV 16 or 18 positivity diagnosis pre-LLETZ was found to be a statistically significant risk factor for persistent disease. It has been described that HPV 16 and 18 are detected in 71% of invasive cervical cancers and HPV 16, 18, as well as 45 are found in 94% of cervical adenocarcinomas, which could be an appropriate argument to perform an ECC at LLETZ in those particular cases [47]. In this study, one of the two cases, in which the ECC led to an altered treatment strategy, was diagnosed with invasive cancer while having an HPV 16 infection. Moreover, patients with high-grade lesions that are not fully visible at a colposcopy might also benefit from the performance of an ECC at LLETZ, since there does not exist a method to ascertain its dispersion within the endocervical canal. On the other hand, it seems reasonable to conclude that patients with no evidence of an endocervical lesion, a visible TZ, and a non-HPV 16/18 infection may not necessarily benefit from an ECC, not only during a colposcopy but also at LLETZ. Nevertheless, the low sensitivity of the ECC (Table 3) in the studied collective indicates that occult (pre)cancerous lesions above the endocervical margin might still be missed despite the use of ECC, which is why further prospective studies need to be conducted in order to identify the specific circumstances where an ECC at LLETZ should not be omitted.

Consequently, the endocervical margin status and therefore the performance of an ECC at LLETZ has a direct impact on further treatment options in the case of invasive carcinoma or AIS and may also be taken into account in women over 50 years of age. It is important to mention that the implementation of universal testing of hr-HPV as a test of cure 6 months post-LLETZ may be difficult, especially in medium- or low-income countries, which would rather use cytologic evaluation [41]. Therefore, an ECC and, in general, the endocervical margin status may have a different value if hr-HPV testing is not available nationwide after LLETZ.

The major strengths of this study are the large number of patients analyzed, the performance of ECC as a routine procedure minimizing a potential selection bias, the application of the descriptive results to current guidelines that allow for clinical implications to be drawn and the rigorous histological review by a small number of specialized pathologists. The retrospective character of the study, the missing data of long-term follow-up including HPV status, and the relatively low number of patients with AIS included are considered as limitations. 

## 5. Conclusions

An ECC performed as a routine procedure at the completion of LLETZ yielded additional diagnostic information in 1.7% of the cases analyzed. A negative ECC yielded a low NPV of 43.8% for the detection of residual disease, which raises the question of whether the performance of an ECC at the time of LLETZ should be performed routinely. Beyond that, current guidelines increasingly support the performance of an HPV test six months after LLETZ as an indicator of treatment success, irrespective of the margin status, which further reduces the clinical importance of the ECC subsequent to the LLETZ for two cases (0.2%) in this study. 

## Figures and Tables

**Table 1:** Basic characteristics.

ECC at The Completion of LLETZ (n = 1121)					
Age, years ^1^					36 (31–44)
Negative margins ^2^					835 (74.5)
Positive margins ^2^					276 (24.5)
			endocervical ^2^		168/276 (60.9)
			ectocervical ^2^		108/276 (39.1)
Margin status non-evaluable ^2^					10 (0.9)
ECC LSIL/HSIL/AIS ^2^					49 (4.4)
		Positive margins ^2^			36/49 (73.5)
		Negative margins ^2^			12/49 (24.5)
		Margin status non-evaluable ^2^			1/49 (2.0)
ECC microinvasive cancer ^2^					1/1121 (0.1)
Immediate surgical treatment ^2^					68/1121 (6.1)
	re-LLETZ ^2^				34/68 (50.0)
	Hysterectomy ^2^				34/68 (50.0)
HPV 16 ^2^					463/1009 (45.9)
HPV 18 ^2^					258/1009 (23.0)
Non-HPV 16/18 ^2^					288/1009 (28.5)
TZ type ^2^				1	600/1036 (57.9)
				2	238/1036 (23.0)
				3	198/10,036 (19.1)
Histological diagnosis LLETZ ^2^				negative	85 (7.6)
				LSIL	117 (10.4)
				HSIL	883 (78.8)
				AIS	36 (3.2)

Data are presented as ^1^ median (interquartile range) or ^2^ number (frequencies). Abbreviations used: ECC, endocervical curettage; (re-)LLETZ, (repeat) large loop excision of the transformation zone; LSIL, low-grade squamous lesion; HSIL, high-grade squamous lesion; AIS, adenocarcinoma in situ; HPV, human papillomavirus; TZ, transformation zone.

**Table 2:** Histological distribution of ECC and LLETZ specimens.

	n = 1121			ECC						Follow Up	
L L E T Z	**Histological Result**			**Negative**	**Inadequate**	**LSIL**	**HSIL**	**AIS**	**CA**	**Additional Diagnostic Information**	**Impact on Therapeutic Approach ^1^**
			N (%)	620 (100)	451 (100)	15 (100)	33 (100)	1 (100)	1 (100)	19/1121 (1.7)	2/1121 (0.2)
	Negative		85 (7.6)	46 (7.4)	38 (8.4)	-	1 (3.0)	-	-	1 (5.3)	1 (50.0)
	LSIL		117 (10.4)	74 (11.9)	39 (8.6)	4 (26.8)	-	-	-		-
		Negative margins	106 (90.6)	69 (93.2)	35 (89.7)	2 (50.0)	-	-	-	2 (10.5)	-
		Positive endocervical margin	8 (6.8)	4 (5.4)	2 (5.1)	2 (50.0)	-	-	-		-
		Positive ectocervical margin	3 (2.6)	1 (1.4)	2 (5.1)		-	-	-		-
	HSIL		883 (78.8)	477 (76.9)	363 (80.5)	11 (73.3)	31 (94.0)	-	1 (100)		
		Negative margins	626 (70.9)	347 (72.7)	271 (74.7)	3 (27.3)	5 (16.1)	-	-	8 (42.1)	-
		Positive endocervical margin	152 (17.2)	71 (14.9)	53 (14.6)	4 (36.4)	23 (74.2)	-	1 (100)	1 (5.3)	1 (50.0)
		Positive ectocervical margin	96 (10.9)	54 (11.3)	36 (10.0)	4 (36.4)	2 (6.5)	-	-	6 (31.6)	-
		Margin status non-evaluable	9 (1.0)	5 (1.1)	3 (0.8)	-	1 (3.2)	-	-	1 (5.3)	-
	AIS		36 (3.2)	23 (3.7)	11 (2.4)	-	1 (3.0)	1 (100)	-		-
		Negative margins	18 (50.0)	14 (60.9)	4 (36.4)	-	-	-	-		-
		Positive endocervical margin	8 (22.2)	4 (17.4)	2 (18.2)	-	1 (100)	1 (100)	-		-
		Positive ectocervical margin	9 (25.0)	5 (21.7)	4 (36.4)	-	-	-	-		-
		Margin status non-evaluable	1 (2.8)	-	1 (0.9)	-	-	-	-		-

Abbreviations used: ECC, endocervical curettage; LLETZ, large loop excision of the transformation zone; LSIL, low-grade squamous lesion; HSIL, high-grade squamous lesion; AIS, adenocarcinoma in situ; CA, invasive cancer. ^1^ according to current national guidelines. Data are presented as numbers and frequencies.

**Table 3:** Prognostic parameters for persistent disease.

Immediate Surgical Treatment (n = 68)	ECC	Margin Status	Endocervical Margin Status
Sensitivity ^1^	35.7	90.5	78.6
Specificity ^1^	80.7	19.2	34.6
PPV ^1^	75.0	64.4	66.0
NPV ^1^	43.8	55.6	50.0

Data are presented as ^1^ percentages (%). Abbreviations used are as follows: ECC, endocervical curettage; PPV, positive predictive value; NPV, negative predictive value.

**Table 4:** Bivariate analysis for detection of persistent disease (n = 68).

Variable	OR (95% Confidence Interval)	*p*-Value
Histologic result of LLETZ		
HSIL LSIL	2.75 (0.82–9.18) Ref.	-
Endocervical margin		
Positive Negative	1.55 (0.37–6.55) Ref.	-
HPV status		
HPV 16/18 HPV non 16/18	4.80 (1.11–20.76) Ref.	*0.026*
ECC at LLETZ		
Positive Negative	2.33 (0.73–7.46) Ref.	-

Significant *p*-values are provided in italics. Abbreviations used are as follows: OR, odds ratio; Ref., Reference value; LLETZ, large loop excision of the transformation zone; LSIL, low-grade squamous lesion; HSIL, high-grade squamous lesion; HPV, human papillomavirus; ECC, endocervical curettage.

## Data Availability

The data presented in this study is available on request from the corresponding author.
